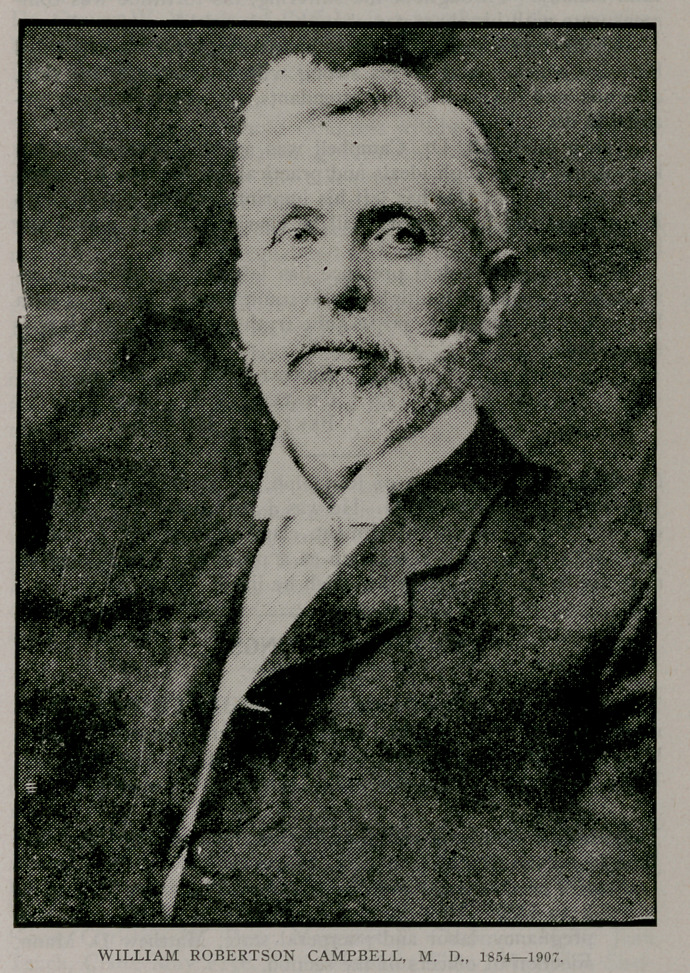# William Robertson Campbell

**Published:** 1907-07

**Authors:** Ernest Wende, Charles G. Stockton, Benjamin H. Grove


### OBITUARY.

### William Robertson Campbell.

AT the thirty-second annual meeting of the Alumni association of the medical department of the University of Buffalo, the death of the president of the association, Dr. William R. Campbell of Niagara Falls, was announced by the chair, who appointed as a committee to prepare a memorial, Drs. Ernest Wende, Charles G. Stockton, and Benjamin H. Grove. Subsequently the following memorial was reported by the committee, which was unanimously adopted by a rising vote.

IN MEMORIAM.

Dr. William Robertson Campbell, of Niagara Falls, entered into rest April 9, 1907, after an illness of a few months. His brief career was, from its incipiency, one of fulness and activity, and his early demise was such as not only to cause the deepest grief, but to invite attention and reflection.

He was born in New York City, April 7, 1854, but his boyhood was passed in Canada where he received his academic education. Early in life, at St. Catherines and Drummondville, he was engaged in commercial pursuits, but his taste and instinct soon caused him to abandon them. The university called him; he wisely obeyed; and the practice of medicine became his chosen profession. His name occurs on the commencement day program, February 25, 1880, with a thesis on "Dropsy." After receiving his degree, he located at Niagara Falls and soon associated himself with Dr. Gardener C. Clark, which relation, full of self-sacrifice and of answering affection, continued up to the latter's death a few years ago. His medical career was distinctively successful, and he applied himself to it in conformity with the highest ideals and most advanced standards. He was a member of the leading societies—president of the Niagara Falls Academy of Medicine; member of the Buffalo Academy of Medicine; for many years, a curator of the University; and, but for his untimely death, as our presiding officer he would have augmented the dignity, the energy, and the prosperous impulse of this eventful occasion. He found his duty, likewise, in the service of the state, serving as Assistant Surgeon to the 42d Separate Company from which he was promoted to Assistant Surgeon of the 1st Batallion of the New York State National Guard.

His private practice was large, and was characterised by skill of high order, and his sympathy and benevolent consideration of the poor, the humble, and the suffering was as generous as it was sincere. The impression made upon all who knew him was that of the *true* physician. The regularity and persistency with which he prosecuted his calling; his extremely modest and unassuming bearing; his cordial helpfulness and kindliness to all who consulted him; his entirely unselfish nature; and the absolute purity of his life and motives were characteristics which marked his whole course.

In man, there are regions more fertile and profound than those of intelligence and reason, and these were manifested not only by the qualities already referred to but by a sense of justice and deep feeling for humanity; and the compassion which pervaded his entire make-up was exceptionally sensitive and responsive to the finer instincts, and extended conspicuously to the love of dumb animals. He was an active member of the Society for the Prevention of Cruelty to Animals. The dumb animals had, in him, a sympathetic friend who felt and thought for them. Compassion with him meant action; and, as has been stated in the press, could they but speak, how deep and profound would be their expression of gratitude.

The noble thoughts that come to all men,—"thoughts that pass across the heart like great white birds,"—were not dismissed by him; on the contrary, they found expression in action and deeds which marked his gentler disposition. His life was one of earnestness; not a day was trivial; and it was part of him, and he lived in it, strengthening his ideals to the ultimate good of his fellow-man in whom he inspired more than ordinary affection, as well as esteem. "His patients loved him," is often said of medical men. In his case, it could be said "it was a self-evident fact."

As a citizen, he enjoyed the confidence of his fellows, and contributed largely to the progress of his city. His energy and many sided ability enabled him to give considerable time to other interests. He was foremost in advancing the industrial welfare of the locality; was made president of the Niagara County Home Telephone Company; a director in the Union Long Distance Telephone Company; president of the New York State Independent Telephone Association; president of the Citizens Lumber Com-

WILLIAM ROBERTSON CAMPBELL, M. D., 1854—1907.

pany; and besides which he was the proprietor of the Niagara Pharmacy. In all these, his personality was felt, and success followed his endeavor.

His illness, which began in December, 1906, determined an operation which revealed what had been predicted, a necessarily fatal malady. Throughout the suffering, his fortitude was commensurate with his character, though he loved life as few did.

A pathetic incident with it was his desire to live his fifty-third birthday which, owing to his strong will, and through the skill, affection and devotion of his attendants, he was fortunately enabled to do.

Taken all in all, Dr. Campbell was a noble personality; his keen moral sense, high ideals and practical sympathetic humanity were part of him. He has left his imprint upon all that came to him in life, and his memory will be cherished by all who knew him.

Our friend's physical presence has left us, but his spirit remains reinforced and multiplied. So could be truly said, that with him it was not enough to possess the truth but that the truth possessed him.

Ernest Wende, *Chairman,*
Charles G. Stockton,
Benjamin H. Grove.

## Figures and Tables

**Figure f1:**